# Pragmatic Use of Standard Outcomes Measures and Data Collection in Adult Day Services

**DOI:** 10.1093/geroni/igaf122.1710

**Published:** 2025-12-31

**Authors:** William Zagorski

**Affiliations:** American Senior Care Centers, Inc., Nashville, Tennessee, United States

## Abstract

The National Adult Day Services Association (NADSA) represents the growing Adult Day Services (ADS) industry, with over 5,000 providers nationwide. To advance the quality of care in ADS, NADSA has established standardized outcome measures, published in the Journal of Applied Gerontology in 2018. Through an innovative public-private collaboration, NADSA has since implemented a secure, national data entry portal to systematically track these outcomes. This dataset represents 40 ADS providers across 11 states, with the capacity to scale to 25,000 participants nationwide. Preliminary results from n = 995 participants across 27 ADS sites indicate high acuity among attendees. Of those assessed, 42% were at high fall risk (Hendrich II), 36% required ambulatory assistive devices, and 77% had high nutritional risk (DETERMINE Checklist). Dementia diagnoses were prevalent (78% per Saint Louis University Mental Status-SLUMS), with significant functional dependence—96% being dependent on assistance with instrumental activities of daily living (iADLs) (Lawton), and 65% with activities of daily living (ADLs) (Katz). Additionally, 24% exhibited signs of geriatric depression (Geriatric Depression Screening-GDS-15). This session will provide insight into NADSA’s evidence-based measures and the largest ADS-specific outcomes dataset in the nation, including change over time in functional status, and health outcomes (results pending). The data are available to academic and institutional partners to advance research on ADS benefits for participants, caregivers, payers, and communities. Discussion will focus on how to leverage this novel dataset to improve standards of care in ADS.

